# Editorial: Challenges in inflammatory bowel disease: current, future and unmet needs, volume II

**DOI:** 10.3389/fmed.2023.1326126

**Published:** 2023-11-13

**Authors:** Antonietta Gerarda Gravina, Raffaele Pellegrino, Giorgia Bodini

**Affiliations:** ^1^Hepatogastroenterology Division, Department of Precision Medicine, University of Campania Luigi Vanvitelli, Naples, Italy; ^2^Gastrointestinal Unit, Department of Internal Medicine, University of Genoa, Genoa, Italy

**Keywords:** inflammatory bowel disease, Crohn's disease, ulcerative colitis, unmet needs, treat-to-target

## Targeting the approach to inflammatory bowel diseases (IBD)

Following up on last year's Research Topic, this year's topic of the same name also provided new and exciting insights into the unexpected needs of IBD (1).

IBD show management and epidemiology that are affected by regional differences. Indeed, it is helpful to focus the research on uncovering these differences to improve disease management.

Karami et al. carried out an interesting annual prospective study by weighing, in Iran, the health-related quality of life (i.e., HRQoL) of patients with moderate to severe Crohn's disease (CD) on biologic drug therapy (i.e., infliximab and adalimumab) in 222 patients. Since such a study was also conducted during the coronavirus pandemic (i.e., COVID-19), part of the work was done by online questionnaires and WhatsApp to reach patients with difficulties participating in person. The authors employed the EuroQol five-dimensional three-level questionnaire and the Inflammatory Bowel Disease questionnaire-short form 9. At multivariate analysis, having other chronic non-IBD comorbidities and being treated with adalimumab were predictors of higher HRQoL. Unemployment and other chronic comorbidities weighed negatively on HRQoL. Online questionnaires during the COVID-19 pandemic mainly considered different research-related outcomes in patients with IBD (e.g., therapeutic adherence, COVID-19 vaccine-related adverse events, psychological distress, and similar) (2–6).

Keskin et al. instead weighed the effectiveness of the DETAIL questionnaire for routinely identifying axial and peripheral arthropathy in patients with IBD in nearly three hundred patients. Patients with a positive questionnaire received a rheumatological examination. The questionnaire identified new rheumatological disorders in 8.2% of patients with ongoing treatment changes.

Evaluating this evidence, we must underline that the research trend in IBD has a significant component evolving toward personalized medicine, a strategy mainly followed in other chronic disorders. Guo et al. wrote an interesting review focusing on this area.

The association of IBD with extra-intestinal manifestations requires more complex treatment to manage both conditions (7). He et al. provided an interesting review summarizing new findings on extra-intestinal cutaneous manifestations associated with IBD with a particular pathophysiological focus.

## Research in IBD has been particularly active for decades with the emergence of even genome-wide association studies

Bibliometric analyses are gradually emerging in the IBD research landscape as a tool for assessing the impact and directions of medical research in certain areas (8). In the context of precision medicine, the omics sciences are gradually evolving to incorporate all the techniques of genomics, transcriptomics, epigenomics, proteomics, metabolomics and the like (9, 10). This is the case with the work of Zhang et al. who analyzed research outputs on ulcerative colitis and omics over the past two decades. From their data, about 22.8% of the research came from the United States of America, that leading nation. The main research elements ranged from identifying genomic, transcriptomic, protein, and metabolic features to identifying genetic etiologies of ulcerative colitis and obtaining diagnostic biomarkers, therapeutic targets, and distinguishing elements with CD. Remaining in the realm of genetically based studies, Ke et al. on the other hand, carried out a bidirectional two-sample Mendelian randomization study as part of a genome-wide association study highlighting that there is a random link between genetically predicted IBD and irritable bowel syndrome (i.e., IBS). An odds ratio of 1.20 for developing IBS was weighted in a sample of over two hundred thousand patients with IBD.

## New therapeutic suggestions for IBD in preclinical studies

Our topic also included a new preclinical study conducted by Lindemann et al. in a dextran sodium sulfate-induced model of colitis in C57BL/6 J mice treated with either cyclosporine A, voclosporin or solvent control. Voclosporin, a new calcineurin inhibitor recently approved for lupus nephritis, resulted in clinical, endoscopic and histological improvement in experimental colitis like cyclosporin A. In detail, voclosporin reduced several pro-inflammatory T cytokines (e.g., interleukins and tumor necrosis factor) while modulating T-cell receptor stimulation.

## Author contributions

AG: Conceptualization, Methodology, Project administration, Supervision, Validation, Writing—original draft, Writing—review & editing. RP: Conceptualization, Methodology, Validation, Writing—original draft, Writing—review & editing. GB: Conceptualization, Methodology, Project administration, Supervision, Validation, Writing—original draft, Writing—review & editing.
